# Localization and density of *Porphyromonas gingivalis* and *Tannerella forsythia* in gingival and subgingival granulation tissues affected by chronic or aggressive periodontitis

**DOI:** 10.1038/s41598-018-27766-7

**Published:** 2018-06-22

**Authors:** G. Amodini Rajakaruna, Mariko Negi, Keisuke Uchida, Masaki Sekine, Asuka Furukawa, Takashi Ito, Daisuke Kobayashi, Yoshimi Suzuki, Takumi Akashi, Makoto Umeda, Walter Meinzer, Yuichi Izumi, Yoshinobu Eishi

**Affiliations:** 1 Department of Periodontology, Graduate School and Faculty of Dentistry, Tokyo Medical and Dental University, Tokyo, 113-8510 Japan; 2 Department of Human Pathology, Graduate School and Faculty of Medicine, Tokyo Medical and Dental University, Tokyo, 113-8510 Japan; 3 Division of Surgical Pathology, Tokyo Medical and Dental University Hospital, Tokyo, 113-8510 Japan; 4 Department of Periodontology, Osaka Dental University, Osaka, 540-0008 Japan; 5 Global Center of Excellence for Tooth and Bone Research, Tokyo Medical and Dental University, Tokyo, 113-8510 Japan; 6 Research Fellow, International Scientific Exchange Fund Program, Japan Dental Association, Tokyo, Japan

## Abstract

*Porphyromonas gingivalis* and *Tannerella forsythia* have been thought to be associated with periodontitis; however comprehensive histopathological localization of bacteria in affected human periodontal tissues is not well documented. In the present study, we examined formalin-fixed paraffin-embedded gingival and subgingival granulation tissues from 71 patients with chronic periodontitis and 11 patients with aggressive periodontitis, using immunohistochemistry with novel monoclonal antibodies specific to *P. gingivalis* or *T. forsythia*, together with quantitative real-time polymerase chain reaction for each bacterial DNA. Immunohistochemisty revealed both bacterial species extracellularly, as aggregates or within bacterial plaque, and intracellularly in stromal inflammatory cells, squamous epithelium, and capillary endothelium of granulation tissue. Combined analysis with the results from polymerase chain reaction suggested that localization and density of *T. forsythia* is closely associated with those of *P. gingivalis*, and that bacterial density is a factor responsible for the cell-invasiveness and tissue-invasiveness of these periodontal bacteria. Detection of these bacteria in the capillary endothelium in some samples suggested possible bacterial translocation into the systemic circulation from inflamed gingival and subgingival granulation tissues. Immunohistochemistry with the novel antibodies showed high specificity and sensitivity, and can be used to locate these periodontal bacteria in routinely-used formalin-fixed paraffin-embedded human tissue sections from systemic locations.

## Introduction

Periodontitis is a multifactorial inflammatory disease (one factor being bacteria biofilm residing on the teeth) causing destruction of surrounding tooth supporting tissues and eventually leading to tooth loss^1^. At the same time periodontitis has been associated with systemic diseases due to increased risk for atherosclerotic cardiovascular diseases^2^, adverse pregnancy outcomes^3^, rheumatoid arthritis^4,5^, aspiration pneumonia^6,7^, and cancer^8,9^. Initial inflammatory reaction occurs as a host response by the human body towards colonizing periodontal bacteria. Among the six closely related groups of periodontal bacterial species recognised by Socransky and Haffajee^10^, *Porphyromonas gingivalis* (PG), *Tannerella forsythia* (TF), and *Treponema denticola* are referred to as the “red complex” and are defined as being the species most strongly associated with periodontitis.

Several molecular techniques have been developed over the last few decades that have allowed us to study periodontal bacteria. These techniques include polymerase chain reaction (PCR)-based methods such as conventional PCR^11–13^, multiplex PCR^14,15^, and real-time PCR^16^, as well as hybridization-based techniques such as oligonucleotide probes^17–19^, whole-genomic checkerboard DNA-DNA hybridization^10^ and DNA microarray^20^. Also, next generation sequencing methods have revealed bacterial community composition in health and periodontitis^21,22^. Despite their accuracy, high sensitivity, and detection specificity, these methods cannot visualise the location of periodontal bacteria in tissue specimens while preserving the tissue architecture for histopathologic examination.

PG and TF have been implicated as microorganisms that are associated with chronic periodontitis in the 1996 Consensus report on Periodontal Diseases^23^. The objectives of the present study were to locate PG and TF using immunohistochemistry (IHC) with novel monoclonal antibodies in formalin-fixed, paraffin-embedded tissue sections of clinically-removed gingival and subgingival tissues affected by chronic or aggressive periodontitis. Extracellular and intracellular localization of these periodontal bacteria in each of the tissue samples was analysed histopathologically with regard to the tissue-invasiveness and cell-invasiveness of each bacterium. The bacterial localization detected by IHC was compared with the bacterial density detected by real-time PCR, as well as with clinical profiles of patients from whom the tissue samples were obtained.

## Results

### Antibody specificity

**Figure 1:**
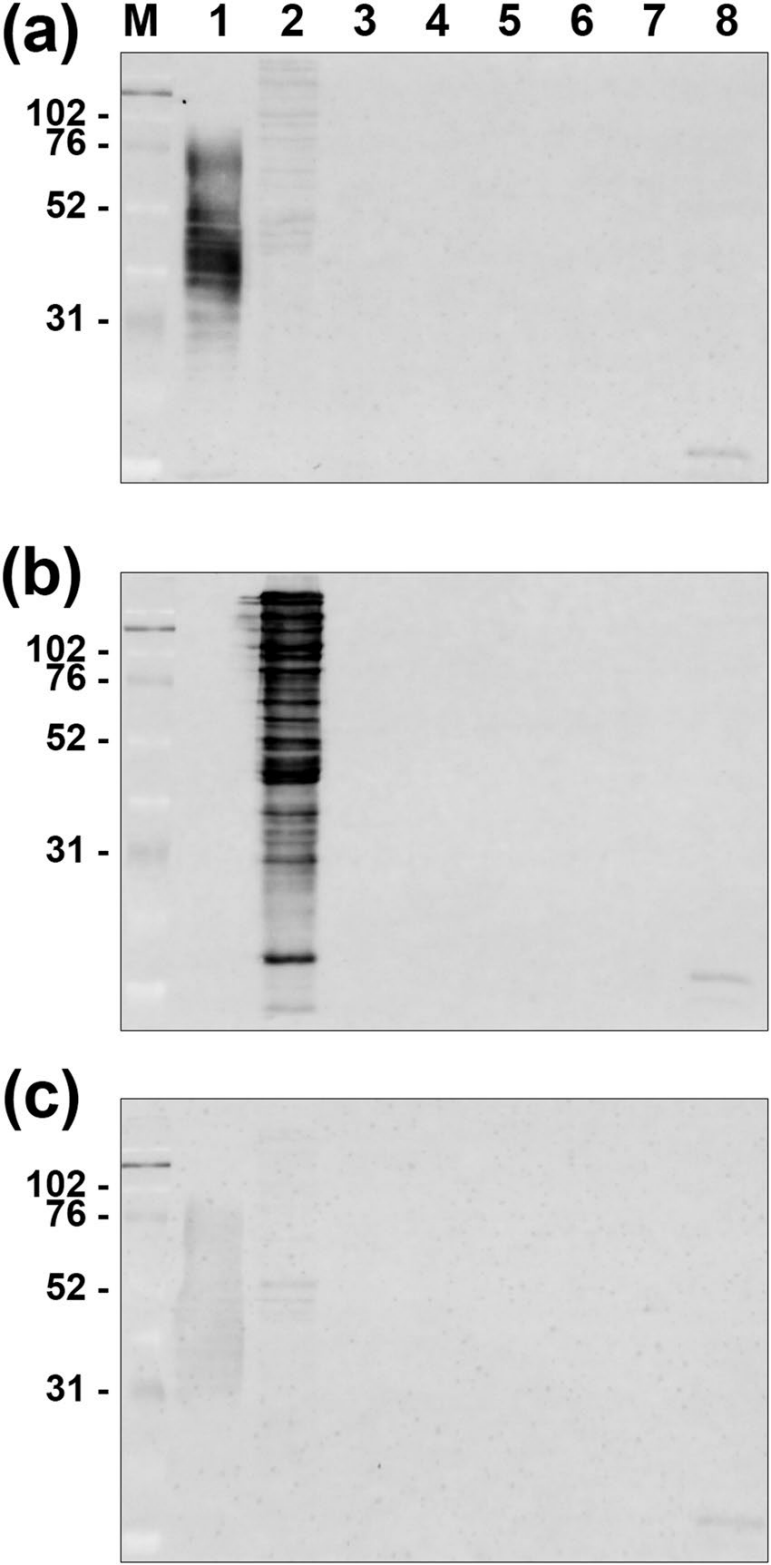
Western blot analysis for reactivity of the anti-PG and anti-TF antibodies. Western blot analysis for reactivity of the anti-PG antibody (**a**; upper column), the anti-TF antibody (**b**; middle column), and the PBS control without primary antibody (**c**; lower column) was performed using sonicated whole-cell lysate. M; marker of molecular weight (molecular mass in kiloDaltons), Lane 1; PG (ATCC 33277), lane 2; TF (ATCC 43037), lane 3; *Aggregatibacter actinomycetemcomitans* (ATCC 43718); lane 4, *Fusobacterium nucleatum* (ATCC 25586), lane 5; *Prevotella intermedia* (ATCC 25611), lane 6; *Prevotella nigrescens* (ATCC 33563), lane 7; *Bacteroides fragilis* (ATCC 25285), and lane 8; *Bacteroides vulgatus* (ATCC 25285). Both the anti-PG and anti-TF antibodies exhibited a ladder pattern of positive bands ranging from 31 to 76 kDa and from 38 to 225 kDa only on the PG and TF lanes (**a**,**b**), respectively. No positive bands were observed in the other lanes or in the PBS control (**c**).

The specificity of the novel monoclonal antibodies is shown in Table 1. By IHC, the anti-PG and anti-TF antibodies gave positive reactions in the Kupffer cells in PG*-* and TF-infected rat liver sections, respectively, and did not cross-react with other bacterial species. The specificity results obtained by IHC were the same when the reactivity was checked using western blot analysis of whole cell bacterial lysates. Western blot analysis with each of the anti-PG and anti-TF antibodies showed a ladder pattern of positive bands ranging from 31 to 76 kiloDaltons (kDa) and from 38 to 225 kDa, respectively. There was no cross-reactivity and no background-reactivity with other examined bacterial species, or with the PBS control (Fig. 1).

**Table 1:** Specificity of the novel monoclonal antibodies prepared for the present study.

Bacterial Species	Strain	Reactivity of			
		Anti-PG antibody		Anti-TF antibody	
		IHC	Western blot	IHC	Western blot
**Oral bacteria**					
*Porphyromonas gingivalis*	ATCC 53978	+	+	−	−
*Porphyromonas gingivalis*	ATCC 33277	+	+	−	−
*Porphyromonas gingivalis*	BAA 1703	+	+	−	−
*Porphyromonas gingivalis*	A7A 128	+	+	−	−
*Tannerella forsythia*	ATCC 43037	−	−	+	+
*Tannerella forsythia*	Clinical Isolate 1	−	−	+	+
*Tannerella forsythia*	Clinical Isolate 2	−	−	+	+
*Tannerella forsythia*	Clinical Isolate 3	−	−	+	+
*Aggregatibacter Actinomycetemcomitans*	ATCC 43718	−	−	−	−
*Fusobacterium nucleatum*	ATCC 25586	−	−	−	−
*Prevotella intermedia*	ATCC 25611	−	−	−	−
*Prevotella nigrescens*	ATCC 33563	−	−	−	−
**Gastrointestinal bacteria**					
*Bacteroides fragilis*	ATCC 25285	−	−	−	−
*Bacteroides vulgatus*	Clinical Isolate	−	−	−	−
*Escherichia coli*	ATCC 25922	−	−	−	−
*Enterococcus faecalis*	ATCC 19433	−	−	−	−
**Respiratory bacteria**					
*Haemophilus influenzae*	ATCC 9334	−	−	−	−
*Legionella pneumophila*	ATCC 33152	−	−	−	−
*Mycobacterium tuberculosis*	ATCC 25177	−	−	−	−
*Streptococcus pneumoniae*	ATCC 33400	−	−	−	−
**Dermatological Bacteria**					
*Propionibacterium acnes*	ATCC 11828	−	−	−	−
*Pseudomonas aeruginosa*	ATCC 27853	−	−	−	−
*Staphylococcus aureus*	ATCC 25923	−	−	−	−
*Streptococcus pyogenes*	JRS 4	−	−	−	−
**Other**					
*Klebsiella pneumoniae*	ATCC 13883	−	−	−	−

### Histologic localization of the bacteria

**Figure 2:**
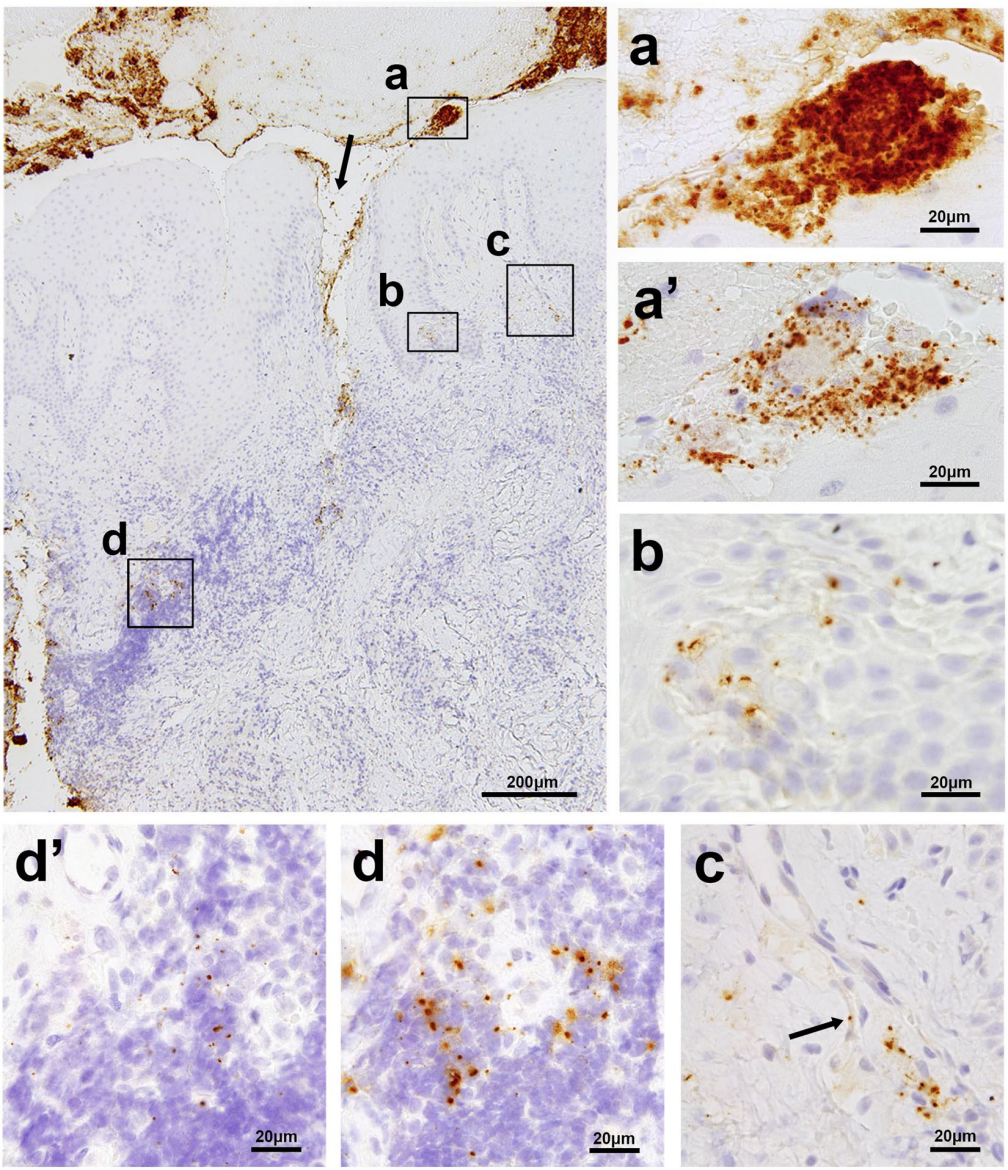
Localization of PG and TF in a representative sample of gingival and subgingival tissue affected by chronic periodontitis. The largest photo shows a low-power view (original magnification, ×100) of the sample immunostained with the anti-TF antibody, representing superficially-located bacterial aggregates or plaques (**a**), bacterial tissue invasion through the disrupted epithelial layer (indicated by the arrow), a cluster of squamous cells with bacteria in the epidermal layer (**b**), a bacterium in a capillary wall of granulation tissue (**c**), and scattered bacterial cells in the area of heavy inflammatory cell infiltration (**d**). The small photos show high-power views (original magnification, ×1000) of the boxed areas indicated by (**a**,**b**,**c** and **d**), including of the photos (**a’** and **d’**) taken from the adjacent sections immunostained with the anti-PG antibody, each corresponding to the area indicated by a and d, respectively. The arrow in photo (**c**) shows a single TF cell located in a capillary wall.

**Figure 3:**
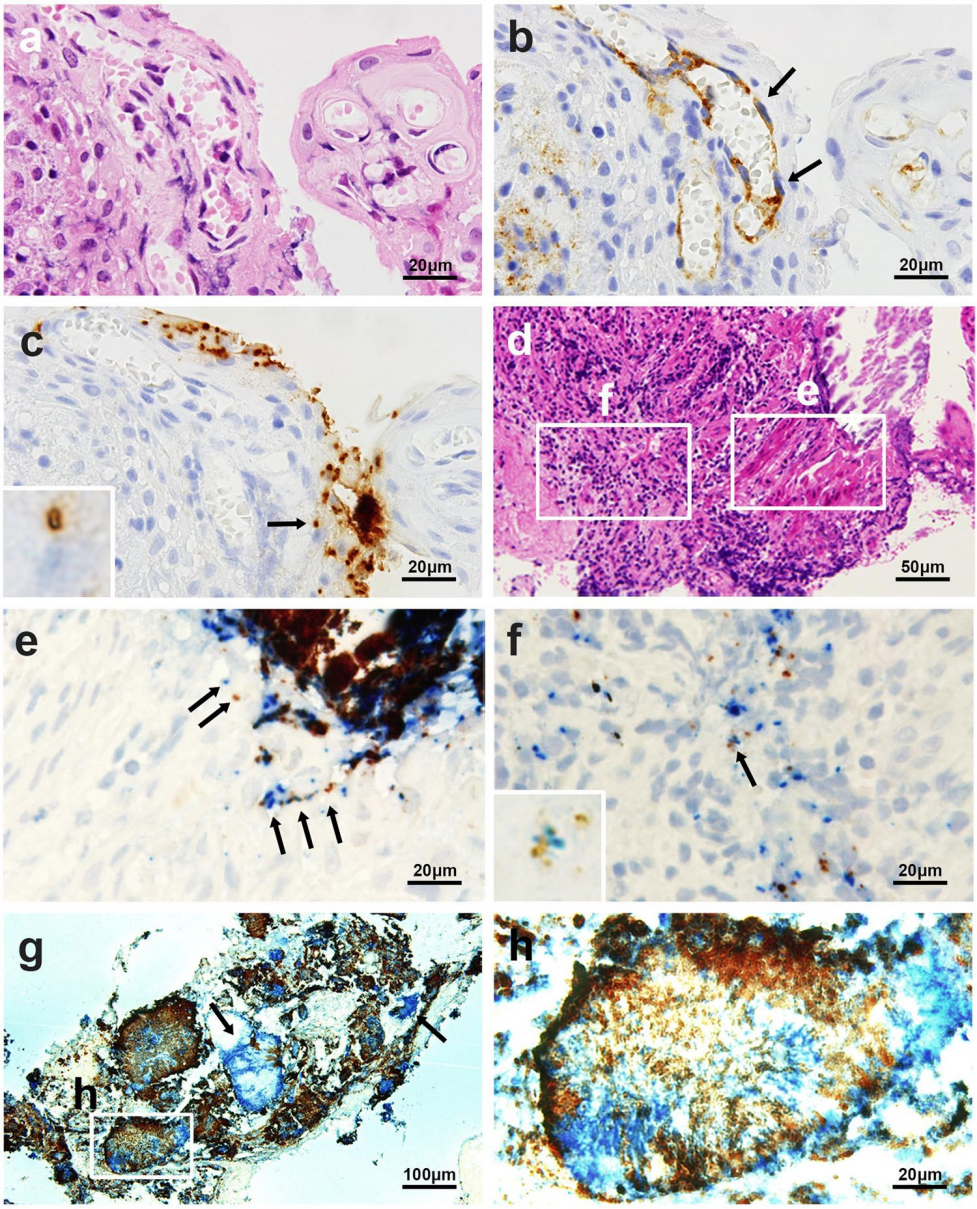
Identification of a bacterium in the capillary endothelium with anti-CD31 antibody and simultaneous identification of PG and TF in identical sections. Three serial sections of a sample with chronic periodontitis with superficial capillary proliferation were stained by hematoxylin and eosin (**a**) and immunostained with the anti-CD31 antibody (**b**) and anti-TF antibody (**c**). The arrows indicate capillary endothelial cells (**b**) containing a single TF bacterial cell in the capillary endothelium (indicated by an arrow and magnified in the inset) (**c**). Two serial sections of a subgingival tissue sample with erosive epidermal layer and subepidermal inflammation were stained by hematoxylin and eosin (**d**) and immunoenzyme-double-stained with anti-PG (brown) and anti-TF (blue) antibodies (**e** and **f**). In the erosive epidermal layer (**e**), one or a few TF and PG cells colocalized in the epithelium (arrows). In the subepidermal inflammatory area (**f**), a few PG cells were found with many TF cells in some of inflammatory cells, and both species cells colocalized in an identical inflammatory cell (indicated by an arrow and magnified in the inset). A section of a sample with many bacterial aggregates and plaques was immunoenzyme double-stained with anti-PG (brown) and anti-TF (blue) antibodies (**g**). Note that most of dark-brown coloured signals are double-positive signals produced by overlapping of blue-coloured TF-positive signals and brown-coloured PG-positive signals. Many plaques or aggregates were intermixed with PG and TF cells, but a few comprised only TF cells (indicated by arrows). A high-power view of a bacterial plaque (**h**) shows the intermixture of both species with a predominant distribution of TF cells in a central core and PG cells in the marginal areas. Original magnifications: a, b, c, e, f, and h, ×1000, d, ×400, and g, ×200.

IHC revealed both bacterial species extracellularly as aggregates or within bacterial plaque and intracellularly in stromal inflammatory cells, squamous epithelium, and capillary endothelium of granulation tissue (Figs 2 and 3). TF and PG cells, when detected together extracellularly, were intermixed within bacterial plaques, mostly with a predominant number of TF cells (Fig. 2a), and a predominant distribution of TF cells in the central core and PG cells in the marginal area (Fig. 3h). Bacterial plaques comprising only TF cells were occasionally observed, but bacterial plaques comprising only PG cells were seldom observed (Fig. 3g).

**Figure 4:**
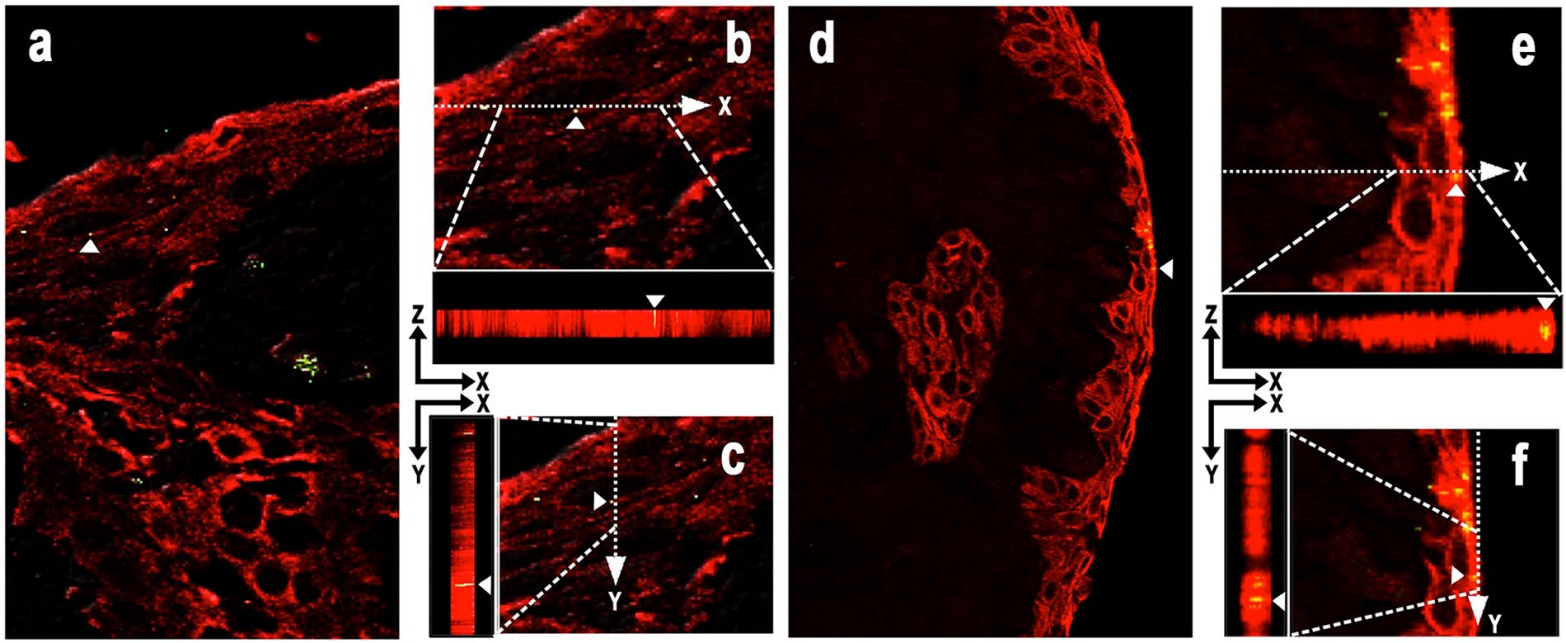
Intraepithelial localization of PG and TF confirmed by immunofluorescence double-staining. Immunofluorescence double-staining of gingival epithelial cells (in red) detected by AE1/AE3 antibody and PG or TF cells (in green) detected by anti-PG antibody (Left; **a**–**c**) or anti-TF antibody (Right; **d**–**f**), respectively. Results of the double-staining are merged in all the pictures. A single double-positive signal (in yellow) indicated by a white arrow head is shown by orthogonal projections constructed in the XZ and YZ planes to confirm the intracytoplasmic localization of these bacteria in mucosal squamous epithelial cells.

Focal clusters of squamous epithelial cells with one or a few bacterial cells intracellularly or intercellularly between desquamative epithelial cells were occasionally observed in the erosive epidermal layer adjacent to superficial bacterial aggregates (Fig. 3e), and sometimes found in deeper epidermal layers (Fig. 2b). Immunofluorescence double-staining signals for PG or TF cells overlapped in the area of the epithelial cytoplasm, which was detected using the anti-cytokeratin antibody (Fig. 4).

PG and TF cells were occasionally clustered in the area of inflammatory cell infiltration and localized in some of the inflammatory cells, which morphologically appeared to be macrophages (Figs 2d and 3f). Intracellular PG or TF cells were rarely detected in the capillary walls of granulation tissue (Fig. 2c). The intracellular bacterial localization in the capillary endothelium was confirmed by IHC with anti-human CD31 antibody (Fig. 3a–c).

### Detection frequency of the bacteria in each location

The extracellular detection and intracellular detection frequencies (in squamous epithelium, stromal inflammatory cell, or capillary endothelium) of PG and TF are shown in Table 2, including the detection frequency by PCR. The extracellular detection frequency by IHC was most similar to the results by PCR. The detection frequency did not differ significantly between chronic and aggressive periodontitis for any location examined. PG and TF were only detected intracellularly in samples that also had extracellular detection of the same species. The frequency of the extracellular and intracellular detection was generally higher for TF than PG. Extracellular PG was only detected in the presence of extracellular TF in 55 (67%) samples, whereas extracellular TF was detected even in the absence of extracellular PG in 15 (18%) samples. Similarly, intracellular PG was only detected in the presence of intracellular TF in 23 (28%) samples, whereas intracellular TF was detected even in the absence of intracellular PG in 20 (24%) samples. Samples without either species detected extracellularly and intracellularly were found in 12 (15%) and 39 (48%) samples, respectively. Intracellular TF and PG in the capillary endothelium was detected in 8 (19%) and 4 (17%) of the 43 and 23 samples with intracellular TF and PG detected, respectively. The endothelial localization of TF was detected in all samples with endothelial PG. All samples with PG or TF detected in the capillary endothelium were accompanied by the same species detected in the stromal inflammatory cells.

**Table 2:** Frequency of immunohistochemical detection of PG and TF in each location and PCR results.

Diagnosis	Number (%) of samples with reactivity		Number (%) of samples with intracellular reactivity located in			Number (%) of positive samples by PCR
Bacterial species detected by IHC and PCR	Extracellular	Intracellular	Stromal inflammatory cells	Squamous epithelium	Capillary endothelium	
**Chronic periodontitis (n = 71)**						
PG and TF	48 (68%)	19 (27%)	14 (20%)	11 (15%)	4 (6%)	53 (75%)
TF only	14 (20%)	19 (27%)	20 (28%)	5 (7%)	4 (6%)	14 (20%)
both negative	9 (13%)	33 (46%)	37 (52%)	55 (77%)	63 (89%)	4 (6%)
**Aggressive periodontitis (n = 11)**						
PG and TF	7 (64%)	4 (36%)	4 (36%)	1 (9%)	0	6 (55%)
TF only	1 (9%)	1 (9%)	1 (9%)	0	0	2 (18%)
both negative	3 (27%)	6 (55%)	6 (55%)	10 (91%)	11 (100%)	3 (27%)
**Total (n = 82)**						
PG and TF	55 (67%)	23 (28%)	18 (22%)	12 (15%)	4 (5%)	59 (72%)
TF only	15 (18%)	20 (24%)	21 (26%)	5 (6%)	4 (5%)	16 (20%)
both negative	12 (15%)	39 (48%)	43 (52%)	65 (79%)	74 (90%)	7 (9%)

### Association between localization and density of the bacteria

**Figure 5:**
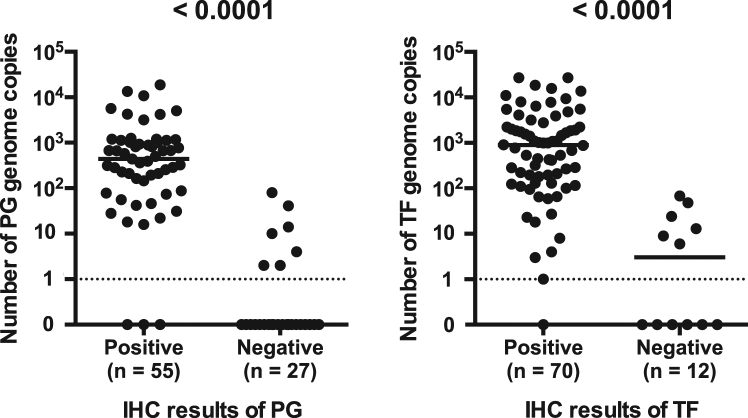
Number of bacterial genome copies of the IHC-positive and IHC-negative samples. Detection status of the bacterium by PCR or IHC for each sample was estimated to be positive when at least one genome copy or one immune-reactive signal was detected by PCR or IHC, respectively. The association between the detection status of the bacterium by PCR and IHC was analysed using Fisher’s exact test.

The number of genome copies for each bacterial species was compared between samples with positive and negative IHC results (Fig. 5). Detection status of the bacterium by IHC and PCR was highly associated for both species (*P* < 0.0001). Positive IHC results of PG and TF were obtained from 52/59 (88%) and 69/75 (92%) of samples with more than one genome copy detected by PCR for the same species, respectively. PCR for TF and PG was negative for one sample and three samples that were IHC-positive for the same species, respectively. In both species, the bacteria number (bacterial density) ranged from 0 to 100 (mean ± SD: PG, 6 ± 17 and TF: 14 ± 22) genome copies in the IHC-negative samples and from 10 to 27,000 (mean ± SD: PG, 1577 ± 3428 and TF: 2997 ± 5618) genome copies in the IHC-positive samples.

Results from IHC detection of extracellular or intracellular localization in either stromal inflammatory cells, squamous epithelium, or capillary endothelium were analysed by comparing with bacterial density of the samples (Table 3). TF density, when detected extracellularly by IHC, was approximately nine times higher in samples with extracellular PG than in those without extracellular PG (*P* = 0.0009). Intracellular detection of PG in stromal inflammatory cells was associated with a higher PG density (*P* = 0.037). Intracellular detection of TF in epithelial cells and in stromal inflammatory cells was associated with the higher TF density (*P* = 0.0021 and 0.0010) followed by a higher PG density (*P* = 0.0031 and 0.013), respectively.

**Table 3:** Association analysis of bacterial localization with bacterial density of samples and with clinical profiles of patients.

Bacterial localization profiles	Number of samples	Number (median) of bacterial genome copies		Mean of probing pocket depth (mm)	Mean age	Sex (male/female)
		PG	TF			
**Total**	82	117	441	6.7	58	31/51
**Extracellular**						
PG and TF	55	440*^a^	1098^*b^	6.9	59^*i^	20/35
TF only	15	0*^a^	124^*b^	6.4	60	8/7
both negative	12	0	3	5.8	49^*i^	3/9
**Intracellular**						
In inflammatory cells						
PG and TF	18	722^*c^	2074	7.2	58	8/10
TF only	21	256^*c^	773	6.9	63	6/15
both negative	43	4	101	6.3	55	17/26
In squamous epithelium						
PG and TF	12	722	2040	6.8	64	5/7
TF only	5	890	4627	6.2	62	1/4
both negative	65	41	444	6.7	56	25/40
In capillary endothelium						
PG and TF	4	2273	4984	7.0	64	2/2
TF only	4	23	8419	7.0	60	1/3
both negative	74	117	369	6.6	57	28/46
**Extracellular PG and**						
PG in inflammatory cells						
positive	18	722^*d^	2074	7.2	58	8/10
negative	37	327^*d^	880	6.7	59	12/25
**PG in epithelium**						
positive	12	722	2040	6.8	64	5/7
negative	43	359	998	6.9	57	15/28
**PG in endothelium**						
positive	4	2273	4984	7.0	64	2/2
negative	51	440	1098	6.9	58	18/33
**Extracellular TF and**						
TF in inflammatory cells						
positive	39	391^*e^	1738^*g^	7.1	60	14/25
negative	31	74^*e^	267^*g^	6.5	57	14/17
TF in epithelium						
positive	17	774^*f^	2236^*h^	6.6	63	6/11
negative	53	88^*f^	531^*h^	6.9	58	22/31
TF in endothelium						
positive	8	159	2500	7.0	62	3/5
negative	62	243	827	6.8	59	25/37

*^a^*P* < 0.0001, *^b^*P* = 0.0009, *^c^*P* = 0.0094, *^d^*P* = 0.037, *^e^*P* = 0.013, *^f^*P* = 0.0031.

*^g^*P* = 0.0010, *^h^*P* = 0.0021, *^i^*P* = 0.0095.

### Association between bacterial localization and clinical profiles

The results obtained from IHC were analysed based on the clinical profiles of the patients (Table 3). Patients with both bacteria detected extracellularly by IHC were older than patients without both bacteria (*P* = 0.0095). The other bacterial localization patterns were not associated with probing pocket depth, age, or sex of the patients. The IHC and PCR results did not differ significantly between chronic and aggressive periodontitis.

## Discussion

This study describes the first histologic localization of PG and TF in the gingival and subgingival tissues affected by chronic or aggressive periodontitis. These periodontal pathogens were located extracellularly on the epithelial surface as aggregates or within bacterial plaques, and intracellularly in the stromal inflammatory cells, squamous epithelium, and capillary endothelium of the granulation tissues. IHC with the novel anti-PG and anti-TF antibodies showed that the antibodies were highly specific and sensitive for detecting the *in situ* localization of these bacteria in formalin-fixed paraffin-embedded tissue sections, and can therefore be generally used for histopathologic examination of the presence of these periodontal bacteria in other systemic organs.

The production of species-specific monoclonal antibodies to detect TF and PG cells in routinely-used formalin-fixed paraffin-embedded tissue sections was successful in the present study with the screening of hybridoma clones by IHC with formalin-fixed paraffin-embedded tissue sections of the liver from rats infected with these target bacteria. The specificity of the antibodies was confirmed by IHC with liver tissue sections of rats infected with other control bacteria as well as confirmed by western blot analysis with bacterial lysates. These species-specific antibodies did not cross-react with other bacterial species within the same order of Bacteroidales (Supplementary Table S1) when used for IHC (Supplementary Fig. S1) and western blot. Species-specific reactivity of the anti-PG antibody was also confirmed by demonstrating no cross-reactivity between PG and *Porphyromonas levii* within the same genus of *Porphyromonas* (Supplementary Fig. S1 and S2). A ladder pattern of positive bands by each of the novel antibodies on western blots suggested that bacterial lipopolysaccharides (LPS) are target antigens of these species-specific antibodies. Indeed, the anti-PG antibody reacted with commercially-available LPS purified from bacterial lysate of PG, showing a ladder pattern of positive bands (Supplementary Fig. S3). LPS, which locates in the outer leaflet of the outer membrane of Gram-negative bacteria, is composed of three parts: lipid A, core sugars, and O-antigen. The O-antigen is a polysaccharide, and the O-specific chain consists of up to 50 repeating oligosaccharide units formed of 2 to 8 monosaccharide components in a highly species-specific manner^24^. Further studies are needed to identify the epitope recognized by each of the novel antibodies. We also attempted to produce an antibody specific to *T. denticola* for use in this study, but our attempts were unsuccessful.

Comparison between the results from IHC and PCR supported the sensitivity of IHC with the anti-PG and anti-TF antibodies. Positive IHC results of PG and TF were obtained from 52/59 (88%) and 69/75 (92%) samples with more than one genome copy detected by PCR for the same species, respectively. The fact that no genome copy was detected by PCR in a few samples with positive IHC results requires some discussion because PCR detection sensitivity is believed to be higher than that of IHC detection. Samples with a few signals (presumably less than 10 signals) detected by IHC may be positive in one tissue section but negative in the other semi-serial sections by IHC due to an uneven distribution of the bacterium across the tissue samples.

IHC of gingival and subgingival tissue samples revealed extracellular PG only when TF was present, whereas TF was detected even in the absence of PG, which is consistent with the results of a previous study using baseline subgingival plaque samples^25^. Moreover, the TF tissue density, when detected by IHC, was remarkably higher in samples with extracellular PG compared to those without extracellular PG. These results suggest that increased levels of extracellular TF produces an environment that promotes the colonization and proliferation of PG, which is consistent with the finding that TF cell extracts stimulate PG growth^26^. The lack of bacterial plaques comprising only PG cells, and the predominant distribution of TF cells in the central core and PG cells in the marginal area of many bacterial plaques, also suggested a PG growth-supporting property of TF. Of particular note, however, even in many samples with extracellular co-localization of PG and TF, intracellular PG was only found in the presence of intracellular TF. On the other hand, intracellular TF was found even in the absence of intracellular PG. This suggests that cell-invasiveness (represented by invasion into squamous epithelium) or tissue-invasiveness (represented by tissue stromal inflammatory cells containing bacteria) of PG may also be supported by the cell-invasiveness or tissue-invasiveness of TF.

Involvement of TF is directly implicated in the development of periodontal disease^27,28^. Indeed, both extracellular and intracellular detection by IHC was more frequent with TF than with PG, and the bacterial density of tissue sections, when detected by IHC, was higher for TF than PG. The concentration of pathogenic bacteria within subgingival plaque may need to surpass a particular threshold in order to induce disease; identifying this threshold may allow the progression of periodontal disease to be predicted^29,30^. In the present study, higher density of TF and PG was associated with detection of the same species in epithelial cells and stromal cells, suggesting that the bacterial density is a major factor responsible for cell-invasiveness and tissue-invasiveness of these periodontal bacteria.

Cell-invasiveness of these periodontal bacteria is associated with cell molecules; such as major fimbriae protein (FimA)^31^ and gingipain^32^ of PG, and leucine-rich surface protein (BspA)^27^ and surface-layer proteins^33^ of TF. Gingipain also damages host cells and connective tissues, which in turn induces apoptosis^32^ and further enhances the ongoing inflammatory reaction leading to periodontal tissue destruction and ulceration in the gingival epithelium (tissue-invasiveness). Otherwise, PG suppresses apoptotic cell death, for intracellular survival, by modulating major apoptotic pathways in gingival epithelial cells^34^.

Although TF is clinically linked with disease, the virulence mechanisms of this organism remain unclear. One possible mechanism of virulence that has been suggested is that TF invades epithelial cells^35–37^. The ability of TF to attach to and invade epithelial cells is promoted by PG and the outer membrane vesicles produced by PG^27^. PG is able to invade the deeper structures of connective tissues via a paracellular pathway by degrading epithelial cell-cell junction complexes^38^. Indeed, we observed the colocalization of PG and TF in the intercellular spaces between desquamative squamous epithelial cells. Consequently, the disrupted epithelial cell barrier caused by intracellular TF infection (cell-invasiveness), supported by intercellular co-localization with PG and other unknown factors, permits extracellular TF and PG invasion into tissue stromal space (tissue-invasiveness). This results in tissue inflammation accompanied by many stromal inflammatory cells that have ingested TF and PG.

Since only one section for each sample was used for IHC detection analysis, the results may be biased especially for intracellular positivity. Nevertheless, samples with any combination of intracellular bacterium detected in stromal inflammatory cells, squamous epithelium, or capillary endothelium of the granulation tissue were always accompanied by extracellular detection of that bacterium. Among them, intracellular detection of bacteria in the capillary endothelium of granulation tissues was always accompanied by intracellular detection of the same species in stromal inflammatory cells. This suggests that the tissue-invading bacteria, many of which are phagocytosed by stromal inflammatory cells, can invade into capillary vessels. IHC-based detection of intracellular bacteria in the capillary endothelium suggested bacterial translocation via the bloodstream. It is generally believed that gingival ulceration in periodontal pockets enables the translocation of bacteria into the systemic circulation, causing bacteremia in patients with periodontitis, which may be an atherogenic stimulus^39^. Tissue-invading periodontal bacteria may translocate to lymph nodes via the lymphatic drainage of the oral cavity^40^, similarly to *Helicobacter pylori* translocating into gastric lymph nodes after invading the gastric mucosa^41^.

Finally, IHC, with the novel antibodies prepared for the present study, allows histopathologic examination to locate these periodontal bacteria in routinely-used formalin-fixed paraffin-embedded tissue sections from surgically-removed specimens to investigate possible translocation of periodontal bacteria to other systemic organs.

## Methods

### Sampling

A total of 82 samples from 82 patients who attended the Periodontics Clinic, Dental Hospital, Faculty of Dentistry, Tokyo Medical and Dental University, were enrolled in the study. The clinical profiles of the patients are shown in Supplementary Table S2 online, and the diagnosis of periodontal disease was made according to the 1999 classification system for periodontal diseases produced by a workshop for clinical periodontitis^42,43^. The mean age was 58 years (SD, 11.9). All patients underwent one round of initial periodontal therapy prior to the surgery. Written informed consent was obtained from all patients after explanation of the purpose of the study. The Ethics Committee of Tokyo Medical and Dental University approved the study (reference 857). All methods were performed in accordance with the relevant guidelines and regulations.

The samples obtained included the deepest non-healing pocket of each tooth with periodontitis, including gingival tissues and subgingival granulation tissues that were curetted following internal bevel incision, during open-flap debridement, Emdogain^TM^ treatment, guided tissue regeneration therapy, apically positioned flap, crown lengthening, or connective tissue graft. Each tissue sample was immediately fixed in 10% buffered formalin for 4 h and then processed using methods for preparing tissues for routine pathologic examination.

### Bacteria culture

Four strains of PG (ATCC 53978, ATCC 33277, BAA-1703, and A7A128) and four strains of TF (ATCC 43037, and 3 clinical isolates) were cultured on Tripticase soy agar^44,45^ plates containing defibrinated horse blood, hemin, and vitamin-K. For TF strain cultures, 15 μl of 20 mg/ml N-acetyl muramic acid solution containing 100 μm sterilised paper disc per Tripticase soy agar plate was added. *Aggregatibacter actinomycetemcomitans*, *Fusobacterium nucleatum*, *Prevotella intermedia*, *Prevotella nigrescens*, *Bacteroides fragilis*, and *Bacteroides vulgatus* were cultured on Anaero Columbia Agar with Rabbit Blood (BD Diagnostic Systems). *Propionibacterium acnes* was cultured on Gifu Anaerobic Medium plates (Nissui Pharm. Co., Ltd.). The above bacterial species were cultured under anaerobic conditions at 35 °C. *Escherichia coli*, *Enterococcus faecalis*, *Streptococcus pneumoniae*, *Pseudomonas aeruginosa*, *Staphylococcus aureus*, and *Klebsiella pneumoniae* were cultured on sheep blood agar M58 (Eiken Chemical Co., Ltd.). *Streptococcus pyogenes* was cultured on Todd-Hewitt agar plates (BD) supplemented with 0.2% yeast extract. *Legionella pneumophila* was cultured on buffered charcoal-yeast extract agar plates (BD). *Mycobacterium tuberculosis* was cultured on Middlebrook 7H11 agar plates (BD). The above bacterial species were cultured under aerobic conditions at 35 °C. *Haemophilus influenza* was cultured on Chocolate II agar plates (BD) under 5% CO_2_ conditions.

### Monoclonal antibody production

Monoclonal antibodies were generated according to the previously described protocol^46^. Briefly, sonicated whole-cell bacterial lysates of PG or TF were used as an immunogen. Hybridoma cell lines were checked with an enzyme-linked immunosorbent assay using bacterial antigens as an immunogen, and screened by IHC with formalin-fixed and paraffin-embedded tissue sections of PG*-* or TF*-*infected rat liver (infected by intravenously injecting 30 mg of heat-killed bacteria into female Sprague–Dawley rats [CLEA Japan] 1 h before killing the rat). Similarly-prepared liver tissue sections of rats injected by each strain of other control bacteria were also examined to confirm the specificity to PG or TF by enzyme IHC with culture supernatant from each hybridoma clone, according to the methods described hereafter.

After selecting the hybridomas that produced a strong reaction specific to each bacterium followed by two rounds of cloning with limiting dilution, a single hybridoma clone was implanted in the intraperitoneal space of severe combined immunodeficiency mice, and ascites was collected and used without further purification. The monoclonal antibodies against PG and TF were named anti-PG antibody and anti-TF antibody, respectively.

### Western Blot analysis

The specificity of the anti-PG and anti-TF antibodies was examined by western blot analysis with four strains of PG, four strains of TF, and many strains of other control bacteria (Table 1). Western blot analysis was performed with whole-cell lysate and commercially-available LPS purified from PG (LPS-PG Ultrapure, InvivoGen, San Diego, CA). Whole-cell lysate of each bacteria were prepared by sonication on ice with an ultrasonic homogenizer (VP-5S ULTRA S homogenizer; Taitec, Koshigaya, Japan) twice at level 6 for 1 min with 100 mg/ml of each bacterial species in phosphate-buffered saline (PBS). Each sample was separated by sodium dodecyl sulfate-polyacrylamide gel electrophoresis using 12% polyacrylamide gel. All samples were transferred electrically to a polyvinylidene difluoride membrane with Mini Trans-Blot cell (Bio-Rad Laboratories). Membranes were blocked overnight at 41 °C with Block Ace (DS Pharma Biomedical Co. Ltd.). The membranes were then incubated for 90 min at room temperature with the appropriately diluted antibodies (1:1000 for both anti-TF and anti-PG antibodies). Membranes were incubated for 30 min with biotinylated rabbit anti-mouse immunoglobulins (DAKO) at a 1:2000 dilution ratio. The membranes were then incubated with FluoroLink^TM^Cy^TM^3-labelled streptavidin (GE Healthcare) for 30 min in the dark with a 1:3000 dilution ratio. Before and after each of the last three steps, the membranes were washed in PBS containing 0.5% Tween-20. Finally, the membranes were washed in PBS before drying them in the dark for 1 h at 37 °C. The dried membranes were viewed using a Bio-Rad BPC-500, Molecular Imager (Quantity One-Analysis Software Version 4.6.9, Bio-Rad). The specificity of both antibodies was evaluated with and without the primary antibody to rule out cross-reactions of whole cell lysates of bacteria with the biotinylated rabbit anti-mouse immunoglobulins.

### Enzyme Immunohistochemistry

The enzyme IHC method used with the anti-PG and anti-TF antibodies was the same as described previously^46^. Histologic sections (4 µm-thick) were cut from formalin-fixed and paraffin-embedded rat liver tissues, and human gingival and subgingival tissue samples, and mounted on silane-coated slides (Muto Pure Chemicals). After the sections were de-paraffinized and rehydrated, they were microwaved (Microwave Processor H2850; Energy Beam Sciences, East Granby, CT, USA) in 10 mmol/l citrate buffer (pH 6.0) for 40 min at 97 °C. The sections were then treated with 3% hydrogen peroxide in methanol for 10 min. The sections were first incubated with normal horse serum (Vectastain Universal Elite ABC Kit; Vector Laboratories) and then incubated overnight at room temperature with one of the appropriately diluted antibodies (anti-PG antibody 1:10,000 or anti-TF antibody 1:128,000) in a humidified chamber. The sections were then incubated for 30 min with biotinylated secondary antibody, followed by 30-min incubation with streptavidin–peroxidase complex (Vectastain Universal Elite ABC Kit), both at room temperature. Before and after each step, the sections were washed in PBS containing 0.5% Tween-20. The signal was developed as a brown reaction product using peroxidase substrate diaminobenzidine (Histofine Simplestain DAB Solution; Nichirei Biosciences Inc.). The staining procedure for IHC with the anti-human CD31 monoclonal antibody (M0823, DAKO) for endothelial cells diluted 1:20 was basically the same except that the microwaving was performed in 10 mmol/l citrate buffer (pH 6.0) for 5 min at 97 °C after deparaffinizing and rehydrating the sections. All specimens were counterstained with Mayer’s hematoxylin. Adjacent histologic sections were also examined with hematoxylin and eosin staining. Since no background signals were produced by the PBS control without primary antibodies in the gingival tissues (Supplementary Fig. S4), the IHC results were considered positive for a single tissue section from each sample when any positive signals were identified in each tissue section or in each type of cell; including squamous epithelium, stromal inflammatory cells, and capillary endothelium. Stained sections were observed and photographed using an OLYMPUS BX-51 research microscope (DP2-BSW application software; Shinjuku, Tokyo, Japan).

### Histologic evaluation

Bacterial aggregates are recognised as clusters of individual bacterial cells on epithelial surfaces and bacterial plaque is recognised as a mass of dense bacterial clusters in free spaces beyond the epithelial surface. PG and TF were considered to be extracellularly localized when at least one positive signal was detected by the anti-PG or anti-TF antibody in the bacterial aggregates or plaques, and intracellularly localized when at least one positive signal was detected by the anti-PG or TF antibody in the cytoplasm of the squamous epithelium or submucosal stromal cells, using serial hematoxylin and eosin-stained sections. We similarly evaluated the intracellular location of PG and TF in the capillary endothelium using serial sections immunostained with anti-human CD31 monoclonal antibody. Three independent observers (MN, TI, and DK) evaluated the histopathologic findings and IHC results. In case of any disagreements between the three observers, the observers re-evaluated the specimens and reached a consensus after discussion.

### Immunoenzyme double-staining

For simultaneous identification of PG and TF in the same tissue section, immunoenzyme double-staining was performed. According to the method described previously^47^, sections were first reacted with the anti-PG antibody using the avidin-biotin-complex method with the VECTASTAIN UNIVERSAL Elite ABC Kit (PK-7200, VECTOR) and the Histofine Simple stain DAB Solution (415174, Nichirei Biosciences Inc.), and microwaved again for 20 min. After these procedures, the sections underwent the secondary reaction with the anti-TF antibody, followed by IHC using a polymer method with Histofine Simple stain AP (M) (414241, Nichirei Biosciences Inc.) and Vector Blue Alkaline Phosphatase Substrate Kit III (SK-5300, Vector).

### Immunofluorescence double-staining

Immunofluorescence double-staining was performed to confirm the intracytoplasmic localization of PG or TF in epithelial cells. According to the method described previously^41^, tissue sections were first reacted with the anti-PG or anti-TF antibody using the avidin-biotin-complex method with fluorescein isothiocyanate-conjugated streptavidin (DAKO), and microwaved again for 20 min. After these procedures, the sections underwent the secondary reaction with anti-human cytokeratin AE1/AE3 antibody (clones:AE1/AE3; DAKO) diluted 1:50, followed by anti-mouse immunoglobulin conjugated with tetra-methylrhodamine isothiocyanate (DAKO). Stained sections were observed and photographed using a FLUOVIEW FV1000-D confocal laser scanning microscope (FV10-ASW1.6 software version; Olympus Co.). Samples showing intracellular positivity were used to acquire consecutive cross-sectional images (XYZ) using the line sequential scanning version of the same microscope. The acquired images were used to produce images in the XY plane, and to confirm the intracellular localization of bacteria, orthogonal projections were produced in the XZ and YZ planes.

### Real-time PCR

Real-time PCR was performed according to the methods described previously^40^. Three serial sections (3 μm-thick) adjacent to the sections used for IHC were sliced from formalin-fixed and paraffin-embedded tissue blocks and placed in sterilised 1.5-ml centrifuge tubes. We used a different microtome blade for each sample to avoid sample-to-sample contamination. The DNA of each section was extracted with a QIAamp for DNA Mini Kit (Qiagen) according to the tissue protocol of the manufacturer. Real-time PCR was performed to amplify fragments of 16S ribosomal RNA of PG and TF. The primers used were PG-F, 5′-GTTCAGCCTGCCGTTG AAAC-3′ and PG-R, 5′-ACCGCTACACCACGAATTCC-3′, and TF-F, 5′-CGACGGAGA GTGAGAGCTTTC T-3′ and TF-R, 5′-GCGCTCGTTATGGCACTTAAG-3′. TaqMan probes were PG-P, 5′- CTTGAGTTCAGCGGCGGCAGG-3′ and TF-P, 5′-CGTCTATGTA GGTGCTGCATGGTTGTCG-3′. The probe was labelled with 6-carboxyfluorescein on the 5′-end and 6-carboxytetramethylrhodamine (TAMRA) on the 3′-end. Real-time PCR mixtures contained 5 µl of template DNA, 100 nM of each primer, 40 nM of the probe, and Absolute QPCR ROX (500 nM) Mix (ABgene) in a total volume of 50 µl. Amplification and detection were performed using a detection system (ABI PRIZM Sequence Detection System; Applied Biosystems) under conditions of 95 °C for 5 min, followed by 50 cycles of 95 °C for 15s and 60 °C for 1 min. The amount of bacterial DNA in each sample was estimated from internal standard samples of serially diluted bacterial DNA (10 ng, 1 ng, 100 pg, and 10 pg) and expressed in terms of the number of bacterial genomes, with 2.5 × 10^9^ Da per genome used in the conversion^48^. Based on the conversion, 10 ng of bacterial DNA equals 2,000,000 copies of 16S rRNA genes. Finally, the results of the quantitative PCR for each sample are expressed as the number of bacterial genomes in the sample. Negative controls without bacterial DNA were included in every PCR; background values were always less than one genome copy. Samples with one or more bacterial genome copy were considered to have positive results.

### Statistical analysis

All analyses were performed using GraphPAD PRISM ver. 6 (GraphPad Software, Inc.). The association of the IHC detection frequency of the bacterium between chronic and aggressive periodontitis, and the association between the detection status of the bacterium by IHC and PCR were analysed using Fisher’s exact test. The Mann–Whitney U test was used to compare the number of genome copies for each bacterial species between samples with both PG and TF, and only TF detected by IHC in each location. Associations of the number of genomic copies for each bacterial species with clinical profiles such as patients’ sex or pocket-probing depth were analysed with the Mann-Whitney U test. In both tests, a *P*-value of less than 0.05 was considered statistically significant.

## Electronic supplementary material

Supplementary Information
