# Supplementary material for: Localization and density of Porphyromonas gingivalis and Tannerella forsythia in gingival and subgingival granulation tissues affected by chronic or aggressive periodontitis
Source: Sci Rep. 2018 Jun 22;8:9507. doi: 10.1038/s41598-018-27766-7 (PMC6014976; doi:10.1038/s41598-018-27766-7)
Supplement: Supplementary file 1 — Supplementary Information [file 41598_2018_27766_MOESM1_ESM.docx]

**Supplementary Information for SREP-17-46013C**

**Localization and density of *Porphyromonas gingivalis* and *Tannerella forsythia* in gingival and subgingival granulation tissues affected by chronic or aggressive periodontitis**

G. Amodini Rajakaruna ^1,5,6¶^, Mariko Negi^2¶^, Keisuke Uchida^3^, Masaki Sekine^3^, Asuka Furukawa^2^, Takashi Ito^2^, Daisuke Kobayashi^2^, Yoshimi Suzuki^2^, Takumi Akashi^3^, Makoto Umeda^4^, Walter Meinzer^1^, Yuichi Izumi^1,5^, and Yoshinobu Eishi^2,3,*^

^1^Department of Periodontology, Graduate School and Faculty of Dentistry, Tokyo Medical and Dental University, Tokyo 113-8510, Japan.

^2^Department of Human Pathology, Graduate School and Faculty of Medicine, Tokyo Medical and Dental University, Tokyo 113-8510, Japan.

^3^Division of Surgical Pathology, Tokyo Medical and Dental University Hospital, Tokyo 113-8510, Japan

^4^Department of Periodontology, Osaka Dental University, Osaka, 540-0008, Japan

^5^Global Center of Excellence for Tooth and Bone Research, Tokyo Medical and Dental University, Tokyo 113-8510, Japan.

^6^ Research Fellow, International Scientific Exchange Fund Program, Japan Dental Association

¶ These authors contributed equally to this work.

**Correspondence:**

Professor Yoshinobu Eishi MD, MedScD, PhD

Department of Human Pathology,

Graduate School and Faculty of Medicine,

Tokyo Medical and Dental University, 1-5-45, Tokyo, 113-8510, Japan. E-mail: [eishi.path@tmd.ac.jp](mailto:eishi.path@tmd.ac.jp)

**Supplementary Table S1 List of bacterial species used in the study to confirm the absence of cross-reactivity of the species-specific anti-PG and anti-TF antibodies with other bacterial species within the same order of Bacteroidales.**

| Phylum | Class | Order | Family | Genus | Species |
| --- | --- | --- | --- | --- | --- |
| Bateroidetes | Bacteriodoa | Bacteroidales | Porphyromonadaceae | *Porphyromonas* | *gingivalis* |
|  |  |  |  | *Porphyromonas* | *levii* |
|  |  |  |  | *Tannerella* | *forsythia* |
|  |  |  | Prevotellaceae | *Prevotella* | *intermedia* |
|  |  |  |  | *Prevotella* | *nigrescens* |
|  |  |  | Bacteroidaceae | *Bacteroides* | *fragilis* |
|  |  |  |  | *Bacteroides* | *vulgatus* |

**Supplementary Table S2 Clinical profiles of the patients.**

| Profiles | Number of samples |
| --- | --- |
| Total number of samples | 82 |
| Age (mean ± SD) | 57.8 ± 11.86 |
| Sex |  |
| Men | 31 |
| Women | 51 |
| Clinical diagnosis |  |
| Chronic Periodontitis | 71 |
| Aggressive Periodontitis | 11 |
| Presence of bleeding on probing |  |
| Bleeding on probing positive | 67 |
| Bleeding on probing negative | 3 |
| Not mentioned | 12 |
| Depth of the deepest pocket (at the site of the surgery) |  |
| Mean ± SD | 6.65 ± 1.80 |
| <6mm | 21 |
| ≥6mm | 61 |

**
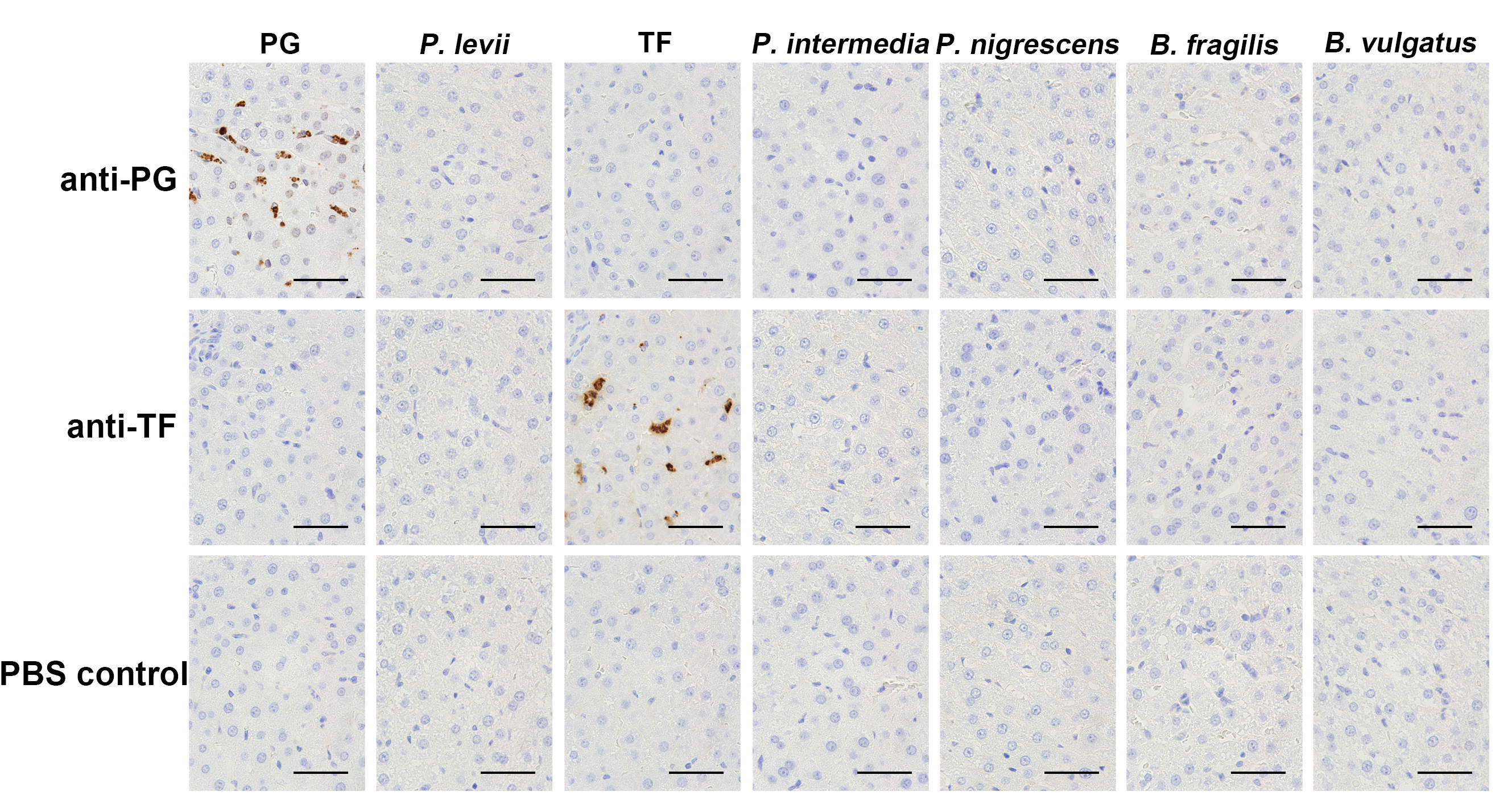
**

**Supplementary Figure S1** **Specificity of the anti-PG and anti-TF antibodies by IHC in bacteria-infected rat liver sections.**

IHC to confirm the specificity of the anti-PG antibody (upper row), anti-TF antibody (middle row) and PBS control without primary antibody (lower row) were performed using rat liver infected with PG, TF and other bacterial species. Scale bar: 40µm


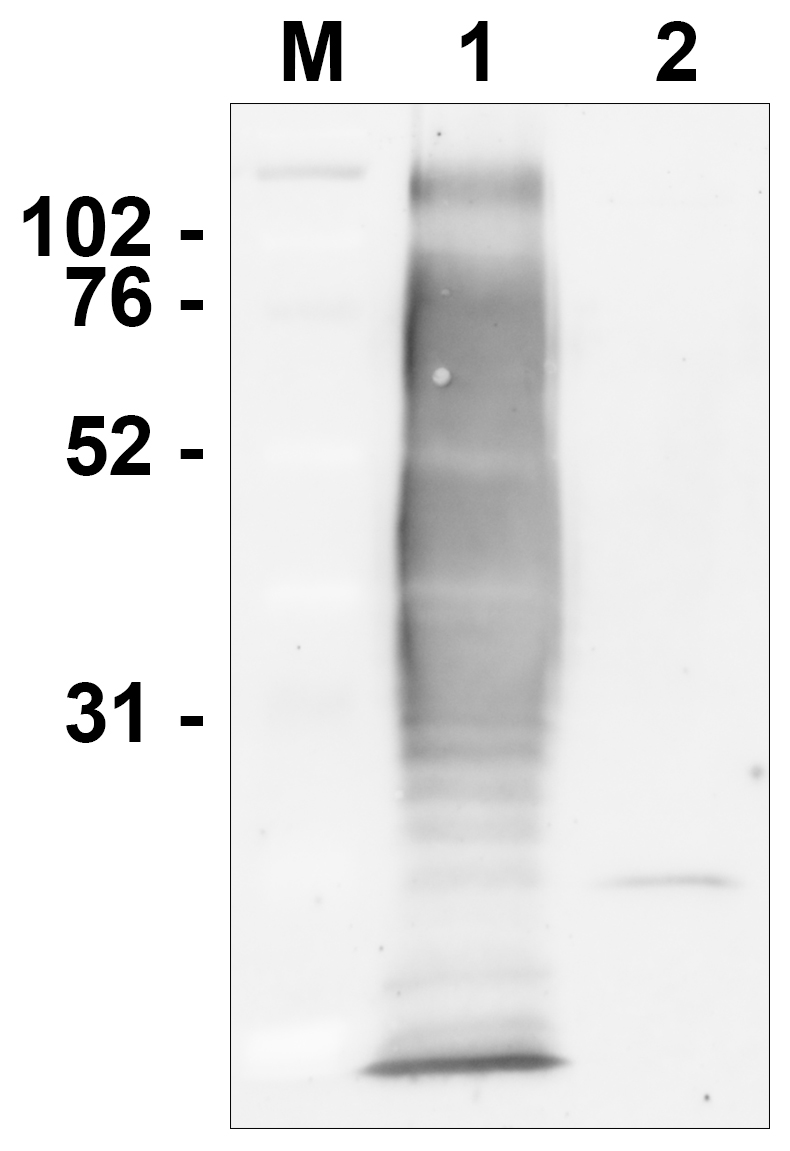


**Supplementary Figure S2 Western blot analysis to demonstrate no cross-reactivity of the anti-PG antibody with *Porphyromonas levii*.**

Western blot analysis to confirm absence of cross-reactivity of the anti-PG antibody with a bacterial species within the same genus of *Porphyromonas* was performed using bacterial lysate of PG and *P. levii*. M; marker of molecular weight (molecular mass in kiloDaltons), Lane 1; bacterial lysate of PG (ATCC 33277), lane 2; bacterial lysate of *P*. *levii* (ATCC 29147). The anti-PG antibody did not cross-react with *P. levii*’s lysate.


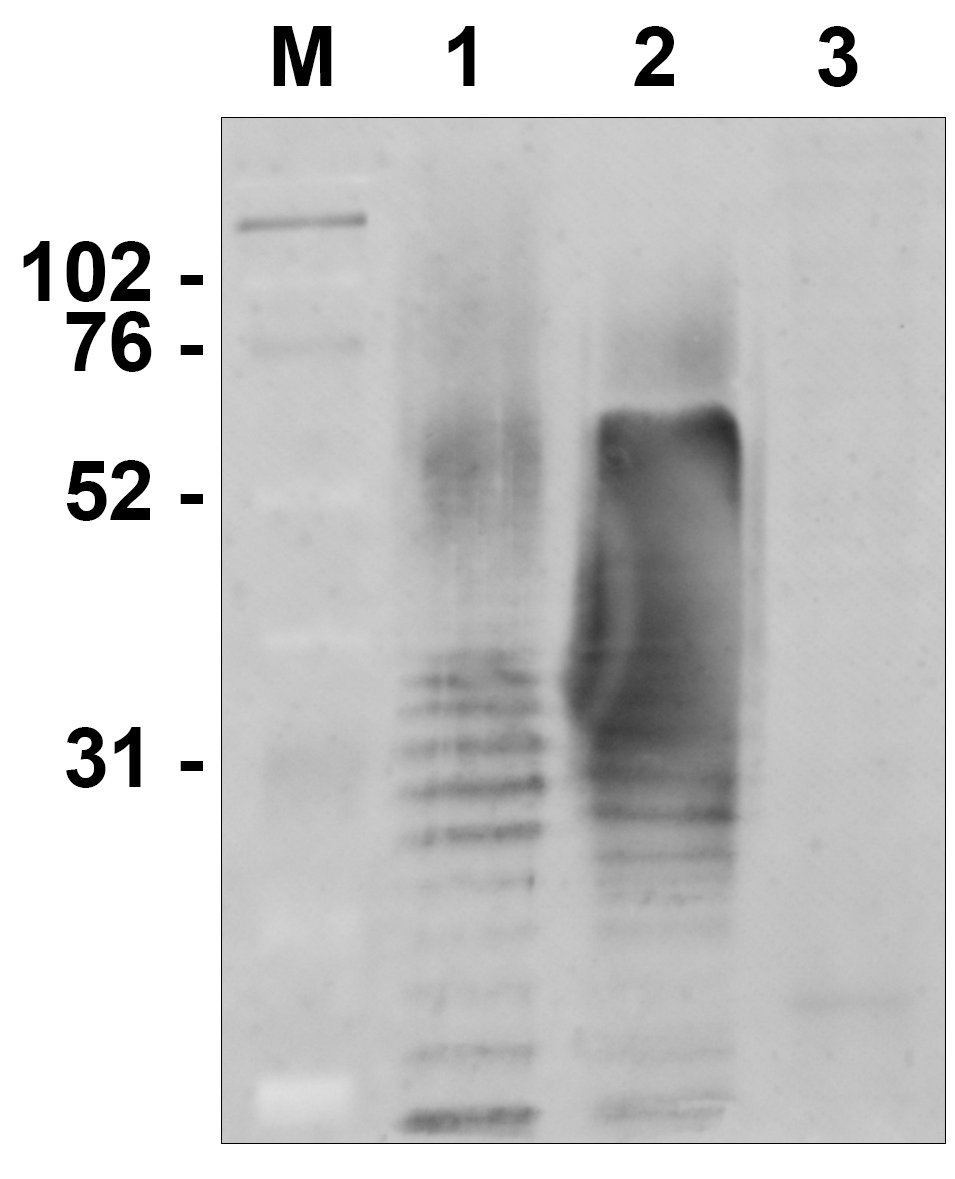


**Supplementary Figure S3 Western blot analysis to confirm the target antigen of the anti-PG antibody.**

Western blot analysis to confirm the target of the anti-PG antibody was performed using commercially-available LPS purified from PG and bacterial lysate of PG and TF. M; marker of molecular weight (molecular mass in kiloDaltons), Lane 1; commercially-available LPS purified from PG, lane 2; bacterial lysate of PG (ATCC 33277), lane 3; bacterial lysate of TF (ATCC 43037). The anti-PG antibody exhibited a ladder pattern of positive bands in both purified LPS and bacterial lysate.


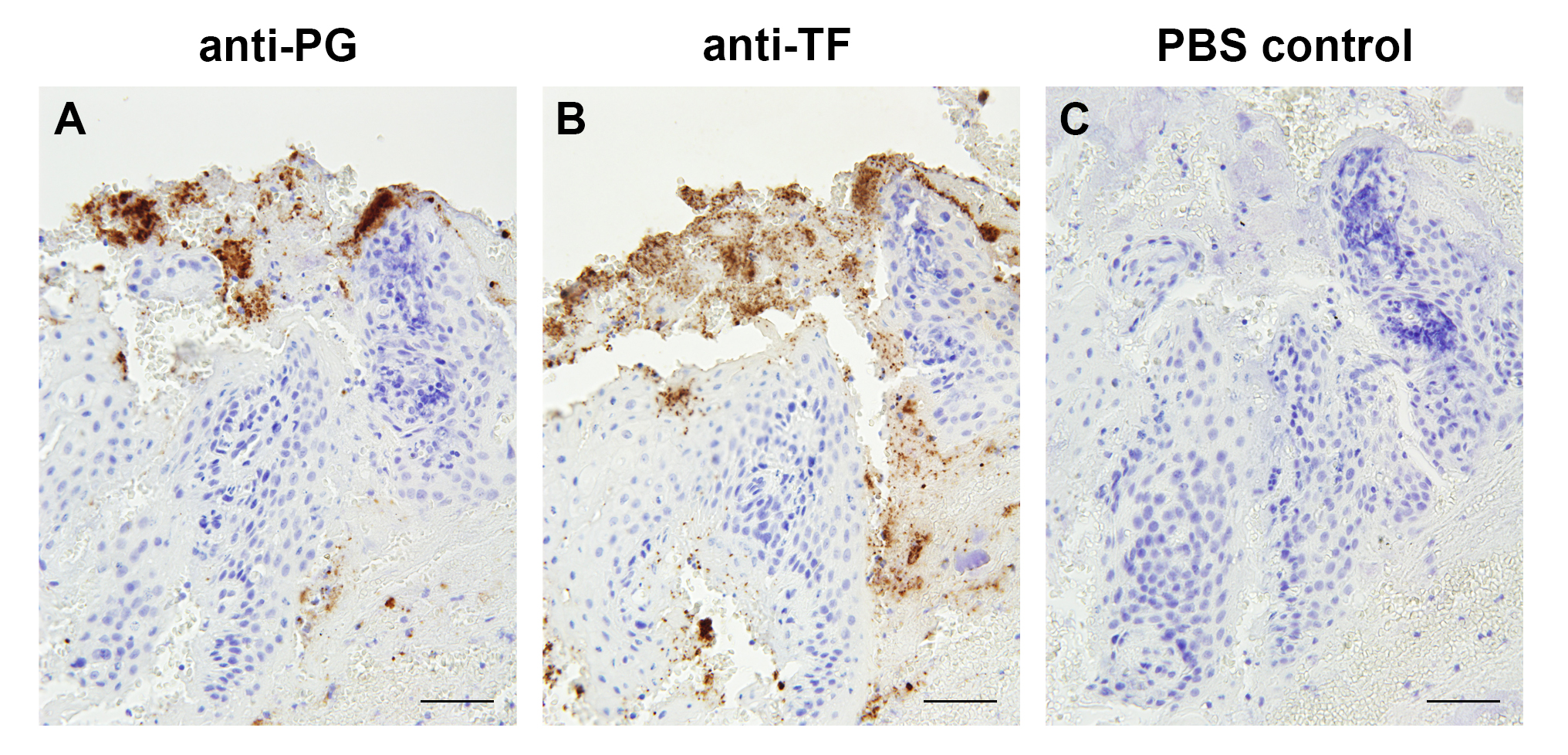


**Supplementary Figure S4** **Total absence of nonspecific background signals by IHC in gingival tissues.**

IHC with the anti-PG antibody (A), anti-TF antibody (B) and PBS control without primary antibodies (C) were performed using semiserial gingival tissue sections of an identical sample. Scale bar: 50µm
